# Lighting Up DNA in the Near-Infrared: An Os(II)–pydppn Complex with Light-Switch Behavior

**DOI:** 10.3390/molecules30244671

**Published:** 2025-12-05

**Authors:** Emanuela Trovato, Salvatore Genovese, Maurilio Galletta, Sebastiano Campagna, Maria Letizia Di Pietro, Fausto Puntoriero

**Affiliations:** 1 Dipartimento di Scienze Chimiche, Biologiche, Farmaceutiche ed Ambientali, Università degli Studi di Messina and Centro Interuniversitario per la Conversione dell’Energia Solare (SOLARCHEM), Via F. Stagno d’Alcontres 31, 98166 Messina, Italy; emanuela.trovato1@unime.it (E.T.); salvatore.genovese@dottorandi.unipg.it (S.G.); campagna@unime.it (S.C.); 2 Dipartimento di Chimica, Biologia e Biotecnologie, University of Perugia, 8, Via dell’Elce di sotto, 06123 Perugia, Italy; 3 CNR-Istituto per la Microelettronica e Microsistemi (IMM), Viale F. Stagno d’Alcontres 31, 98166 Messina, Italy; maurilio.galletta@cnr.it

**Keywords:** DNA light-switch, near-IR luminescence, osmium complex

## Abstract

The osmium(II) polypyridyl complex [Os(tpy)(pydppn)]^2+^ (tpy = 2,2′:6′,2″-terpyridine; pydppn = 3-(pyrid-2′-yl)-4,5,9,16-tetraaza-dibenzo[a,c]naphthacene) was synthesized and characterized to evaluate the effect of an extended planar π-system on photophysical properties and DNA interactions. This complex represents the π-expanded analog of the previously studied [Os(tpy)(pydppz)]^2+^ system. Electrochemical studies revealed a reversible Os(II)/Os(III) oxidation at +0.99 V vs. SCE and five ligand-centered reductions, generally less negative than those of the smaller pydppz analog, consistent with enhanced electron-accepting ability. In acetonitrile, the complex exhibits UV absorption bands at 328 and 473 nm and near-infrared emission at 840 nm, assigned to a long-lived ^3^MLCT state (*τ* = 110 ns, *Φ* = 0.02). Upon titration with calf-thymus DNA, [Os(tpy)(pydppn)]^2+^ shows a pronounced light-switch effect, hypochromism, red-shifted MLCT bands, induced circular dichroism, and an increase in DNA melting temperature (Δ*T*_m_ = 8.9 ± 0.5 °C), consistent with intercalative binding. Viscometric titrations further support intercalation, with a binding constant *K*_B_ ≈ 1.2 × 10^6^ M^−1^. Transient absorption spectroscopy indicates that DNA binding prolongs the excited-state lifetime and modifies vibrational relaxation pathways. These results highlight how π-system extension in Os(II) complexes modulates photophysical behavior and DNA affinity, offering insights for the rational design of NIR-emitting, DNA-targeted luminescent probes and potential phototherapeutic agents.

## 1. Introduction

Transition metal polypyridyl complexes, particularly those of ruthenium(II) and osmium(II), have garnered significant attention in bioinorganic chemistry due to their tunable photophysical properties and their ability to intercalate into DNA, making them valuable tools for DNA sensing and fundamental photochemical studies [1,2]. The design of complexes incorporating extended π-system ligands, such as dipyrido [3,2-a:2′,3′-c]phenazine (dppz) and its derivatives, has been pivotal in enhancing DNA-binding affinity and luminescent properties [3,4,5].

Ruthenium(II) complexes, such as [Ru(bpy)_2_(dppz)]^2+^, display the well-known “light-switch” effect: they are weakly luminescent in aqueous solution, but their emission intensity increases dramatically upon intercalation into DNA due to protection of the excited state from non-radiative decay pathways [6,7,8]. Modifications of the planar ligand framework, including the use of 3-(pyrid-2′-yl)dipyrido-[3,2-a:2′,3′-c]phenazine (hereafter pydppz), expand the π-system and can shift the emission further into the red, improving their utility in bioimaging applications [9,10,11,12]. In particular, [Ru(tpy)(pydppz)]^2+^ has been studied for its DNA light-switch behavior, demonstrating that subtle ligand modifications can significantly influence both DNA-binding affinity and photophysical response [13,14].

The [Ru(tpy)(pydppn)]^2+^ complex, where pydppn is 3-(pyrid-2′-yl)-4,5,9,16-tetraaza-dibenzo[*a*,*c*]naphthacene, represents a further evolution, incorporating a larger planar π-system compared to pydppz. This design enhances intercalation into DNA and modulates the excited-state dynamics [15,16,17]. Compared to [Ru(tpy)(pydppz)]^2+^, [Ru(tpy)(pydppn)]^2+^ displays red-shifted emission and longer-lived excited states while maintaining a pronounced light-switch effect upon DNA binding [16]. Quantitative studies reveal that the binding constant of [Ru(tpy)(pydppn)]^2+^ to calf-thymus DNA is significantly higher than that of [Ru(tpy)(pydppz)]^2+^, indicating the positive contribution of the extended π-system to the intercalative stabilization [16,17,18].

On the other hand, osmium(II) polypyridyl complexes have garnered significant attention due to their unique attributes [19,20]. Compared to their lighter congeners, they often exhibit enhanced spin-orbit coupling effects due to the heavier metal center, so offering complementary advantages due to stronger spin-orbit coupling that can lead to more efficient intersystem crossing, populating long-lived triplet excited states [19,20,21] that are crucial for various applications, including the generation of ROS for PDT [19,20,21,22,23,24]. As we previously reported, the Os(II) analog of [Ru(tpy)(pydppz)]^2+^, namely [Os(tpy)(pydppz)]^2+^, exhibits a red-shifted emission, enhanced photostability, and longer excited-state lifetimes compared to the corresponding Ru(II) complex [25].

**Figure 1:**
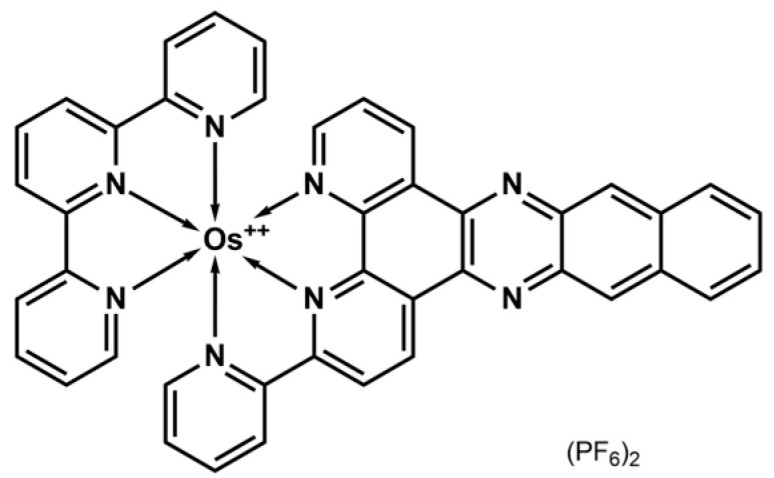
Structural formula of [Os(tpy)(pydppn)]^2+^ (**1**).

In this study, we report the synthesis, comprehensive spectroscopic and electrochemical characterization, and detailed investigation of its interaction with DNA of an osmium(II) complex, [Os(tpy)(pydppn)]^2+^ (hereafter **1**; tpy = 2,2′:6′,2″-terpyridine; pydppn = 3-(pyrid-2′-yl)-4,5,9,16-tetraaza-dibenzo[*a*,*c*]naphthacene) (Figure 1).

This complex represents the π-expanded analog of the previously studied [Os(tpy)(pydppz)]^2+^ system. The primary objective was to elucidate how the presence of this large, extended aromatic surface, particularly when compared to a smaller analogous ligand like pydppz, influences both the intrinsic photophysical properties of the Os(II) center and the complex’s ability to interact with DNA. Understanding these fundamental structure-property relationships is paramount for the rational design and development of next-generation metallo-intercalators with enhanced capabilities as molecular probes and therapeutic agents targeting nucleic acids.

## 2. Results and Discussion

### 2.1. Synthesis and Characterization

The osmium(II) complex, [Os(tpy)(pydppn)]^2+^ (**1**), was synthesized from the precursor [Os(tpy)Cl_3_] following established procedures for Os(II) polypyridyl complexes [25]. The general synthetic strategy involves the substitution of the chloride ligands in [Os(tpy)Cl_3_] by the polypyridyl ligand pydppn under an inert atmosphere and reflux conditions, yielding the desired complex [Os(tpy)(pydppn)]^2+^. Following the reaction, **1** was isolated and purified by chromatography. The stability of **1** in solution was evaluated by monitoring the absorption spectra of the compound in both acetonitrile and buffer solution at temperatures up to 45 °C. No significant changes were observed over the course of the experiment–see Supplementary Materials-indicating that **1** remains stable under these conditions.

### 2.2. Redox Properties

**Figure 2:**
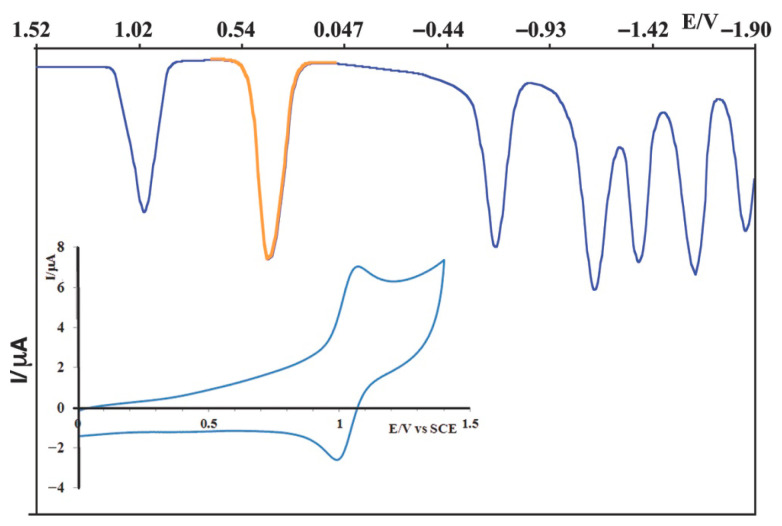
Differential pulse voltammogram of **1** in CH_3_CN ([1] = 500 μM; supporting electrolyte: [TBAPF_6_] = 50 mM; scan rate = 20 mV s^−1^). Inset: Cyclic voltammogram of **1** in CH_3_CN under an inert argon atmosphere (scan rate = 200 mV s^−1^). The ferrocene oxidation wave, used as an internal reference, is highlighted in orange.

The electrochemical behavior of **1** was comprehensively investigated by cyclic voltammetry (CV) and differential pulse voltammetry (DPV) in acetonitrile solution, as shown in Figure 2 and Table 1, which also report the data of [Os(tpy)(pydppz)]^2+^ used as the model (**MOD**).

The measurements were conducted with 100 mM tetrabutylammonium hexafluorophosphate (TBAPF_6_) as the supporting electrolyte, and all reported potentials were referenced against a saturated calomel electrode (SCE).

**1** exhibited a reversible one-electron oxidation process at an anodic potential of +0.99 V vs. SCE. This redox event is unambiguously assigned to the Os(II)/Os(III) couple, representing the oxidation of the central osmium metal ion from its +2 to its +3 oxidation state. The reversibility of this process indicates that the Os(II) complex is electrochemically stable and can undergo a facile one-electron transfer, which is an important consideration for its stability in biological environments where redox processes are common. This oxidation potential is consistent with values reported for analogous Os(II) polypyridyl complexes and for the **MOD**, further supporting its assignment [11,12,25].

In the cathodic region of the voltammogram, five distinct and well-resolved reduction processes were observed. These multiple reduction waves are indicative of the successive acceptance of electrons into the low-lying π∗ antibonding orbitals predominantly localized on the coordinated terpyridine (tpy) and the extended pydppn ligands. The first reduction wave, occurring at the most positive potential among the cathodic processes, is generally associated with the reduction in the most easily reducible ligand. Given the extended π-system of pydppn, its orbitals are expected to be lower in energy and thus more susceptible to reduction.

To understand the specific influence of the pydppn ligand, a comparative analysis with the **MOD** species [25], is mandatory. The pydppz ligand is structurally related to pydppn, but possesses a smaller and less extended aromatic π-system. A significant finding was that the reduction potentials of **1** were generally found to be less negative compared to those of [Os(tpy)(pydppz)]^2+^. This observation is highly informative. A less negative reduction potential implies that the extended π-system of the pydppn ligand in **1** facilitates electron acceptance more readily. The larger, more delocalized π-electron cloud of pydppn provides a more effective sink for additional electron density, making the ligand reductions occur at less energetic potentials. These differences in ligand-based redox behavior are directly relevant to the complex’s capacity to participate in light-induced electron transfer reactions, which are fundamental to the mechanism of action of various photoactive molecules in biological systems [26,27,28].

### 2.3. Spectroscopic and Photophysical Properties

**Figure 3:**
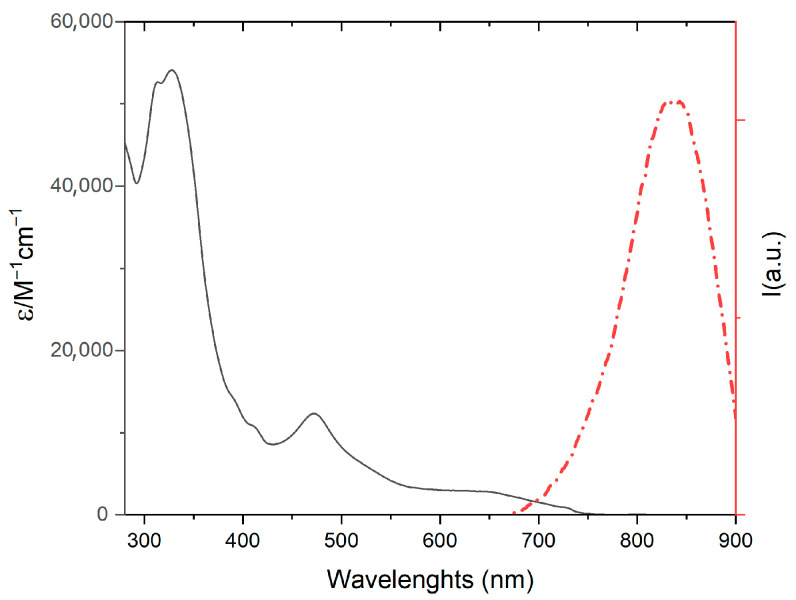
Absorption (black line) and emission (red line) spectra of **1** in CH_3_CN (λ_exc_: 490 nm).

The absorption and emission properties of **1** were thoroughly investigated in acetonitrile solution to elucidate its electronic structure and excited-state behavior, see Figure 3 and Table 2. The absorption spectrum displayed several characteristic bands across the UV-Vis region. In the ultraviolet (UV) region, intense absorption bands were observed around 300 nm and 350 nm. These high-energy bands are unequivocally assigned to spin-allowed ligand-centered (LC) π→π∗ transitions. These transitions originate from electronic excitations primarily localized within the extensive aromatic π-systems of both the tpy and pydppn ligands.

In the visible region, **1** exhibited moderately intense absorption bands, specifically centered in the range 400 ÷ 550 nm., see Figure 3. These lower-energy bands are characteristic of spin-allowed metal-to-ligand charge transfer (MLCT) transitions. In these transitions, an electron is promoted from the d-orbitals of the osmium(II) metal center to the π∗ antibonding orbitals of the coordinated ligands. Given the extended conjugation and likely lower-lying LUMO of the pydppn ligand compared to tpy, these MLCT bands are predominantly attributed to Os(dd) → pydppn(π*) transitions. The presence of these strong absorption features in the visible spectrum indicates that **1** is an efficient light harvester in this therapeutically relevant spectral window.

It is also noteworthy that in the ultraviolet region two intense, partially overlapping bands are observed. By comparison with literature data for structurally related terpyridine and Os(II)–terpyridine complexes, the feature at around 310 nm can be assigned to a ligand-centered (LC) transitions involving the terpyridine chromophore [29,30]. The shoulder-like peak at approximately 320 nm, which overlaps with the former, is plausibly associated with an additional LC transition localized on the more extended “terpyridine-like” fragment of the pydppn ligand [4,25].

At lower energy, around 400 nm, two weak shoulders can be detected. These features are characteristic of complexes containing a phenazine-like N,N-donor unit and can therefore be attributed to LC transitions predominantly involving this portion of the pydppn ligand framework [31].

Regardless of the excitation wavelength, upon photoexcitation, **1** exhibited significant emission in the near-infrared (NIR) region, with an emission maximum observed at 830 nm in acetonitrile, Figure 3. This NIR emission is a highly desirable property for bioimaging applications, as light in this spectral window experiences minimal scattering and absorption by endogenous biological chromophores (e.g., hemoglobin, water) [24,26,27,28], allowing for deeper tissue penetration and reduced autofluorescence interference. The emission is unequivocally attributed to a long-lived ^3^MLCT excited state. The excited-state lifetime (*τ*) was measured to be 110 ns, and the luminescence quantum yield (*Φ*) was determined to be 0.02. It is interesting to note that **1** exhibits both a longer luminescence lifetime and a higher quantum yield compared to the **MOD** complex. This increase in excited-state lifetime can be rationalized by the lower-energy LUMO in the pydppn-containing complex, which enlarges the energy gap with the metal-centered triplet (^3^MC) state. As this ^3^MC state is a known non-radiative decay pathway for the ^3^MLCT state in polypyridine metal complexes, the larger energy separation reduces deactivation, resulting in longer-lived luminescence. The relatively long lifetime of the excited state is a crucial photophysical parameter, as it provides ample time for the excited photosensitizer to interact with its immediate surroundings, including potential biological targets or molecular oxygen, prior to returning to the ground state. The energy of this ^3^MLCT state and its significant lifetime are consistent with properties found in various photoactive metal complexes utilized in imaging and other light-driven processes [26,27,28].

### 2.4. DNA Interaction: The “Light-Switch” Effect

**Figure 4:**
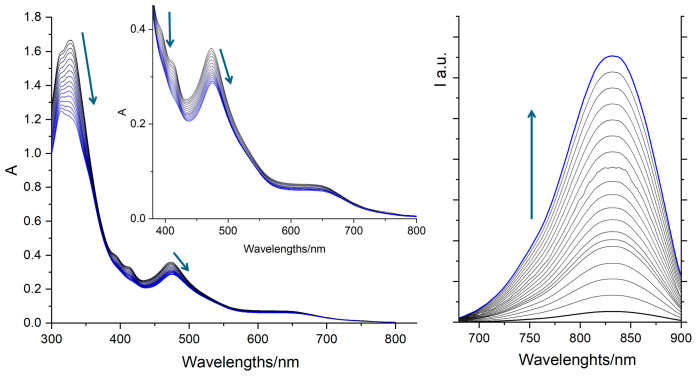
Absorption (**left**) and emission (**right**) spectra of **1** (35 μM) at 25 °C and pH 7 (1 mM phosphate buffer and 57 mM NaCl) upon successive additions of CT-DNA. Excitations have been performed at 550 nm (un isosbestic wavelength). The inset in the left panel shows a zoomed view of the visible portion of the spectra.

The interaction of **1** with calf thymus DNA (CT-DNA), a widely accepted model for double-stranded DNA in eukaryotic cells, was thoroughly investigated using both emission and absorption spectroscopy in aqueous solutions, see Figure 4. These experiments were conducted in 1 mM phosphate buffer, at pH 7.0, containing 57 mM NaCl.

In an aqueous buffer solution without DNA, **1** displayed only very weak intrinsic luminescence. This phenomenon is commonly observed for many transition metal complexes in protic solvents like water and is typical of dppz-like compounds in which efficient non-radiative decay pathways, such as vibrational relaxation involving solvent molecules or rotation of flexible ligand parts, can quench the excited state and dissipate energy as heat, leading to low quantum yields.

However, upon the gradual addition of increasing amounts of CT-DNA to a fixed concentration of **1** (35 μM), a dramatic and significant enhancement in luminescence intensity was observed. This striking phenomenon, where a non- or weakly emissive probe becomes strongly luminescent upon binding to its biological target, is famously known as a “light-switch” effect [1,2,3,4,5,6]. The “light-switch” behavior is a powerful and direct spectroscopic signature of a specific and intimate interaction between the metal complex and DNA. The binding of **1** to DNA likely provides a more rigid and less polar microenvironment for the complex [4,16]. This rigidification restricts the molecular motions and vibrations that contribute to non-radiative deactivation, effectively shielding the complex from interactions with solvent molecules (like water) that would otherwise quench its luminescence. Consequently, the radiative decay pathways become more competitive, leading to a substantial increase in the observed luminescence, causing the complex to “turn on” its light emission when bound to DNA. While the “light-switch” effect has often been regarded as confirmation of DNA intercalation, recent studies have shown that even non-intercalating bimetallic complexes can exhibit this behavior, suggesting that intercalation is not exclusively required for it [32]. For this reason, an in-depth study of **1**’s interaction with DNA was conducted through various spectroscopic and viscometric techniques, which strongly points towards an intercalative mode.

Detailed insights into the binding mode were obtained from spectrophotometric titrations, see Figure 4. When increasing aliquots of concentrated CT-DNA solution were added to a fixed concentration of **1** (35 µM), notable and characteristic changes occurred in the absorption spectrum of the complex. Specifically, the characteristic MLCT absorption bands (in the visible region, around 400 nm and 470 nm) exhibited two key spectral changes. A significant decrease in the absorbance intensity of the MLCT bands was observed. Hypochromism in DNA binding studies typically arises when the electronic transitions of the intercalating molecule are perturbed by strong stacking interactions with the DNA base pairs [5,6,32]. The reduction in absorbance suggests a strong electronic coupling between the chromophore of the complex and the DNA bases, leading to a redistribution of oscillator strength.

Concurrently to this scenario, a distinct red-shift (a shift in the absorption maximum to longer wavelengths) was observed for the MLCT bands. A red-shift generally indicates a stabilization of the excited state of the complex upon binding to DNA. This stabilization occurs because the non-polar, hydrophobic environment within the DNA helix, formed by the stacked base pairs, can provide a more favorable energetic landscape for the excited-state charge transfer of the complex compared to the aqueous solvent [6,7,8,16].

A further spectral variation is observed in the UV region. The change in the intensity ratio of the high-energy bands can be attributed to the different electronic responses of the two ligand frameworks (tpy and pydppn). Intercalation primarily affects the more π-extended pydppn ligand, causing a reduced absorption cross-section and a slight red shift in its intraligand transitions, which predominantly contributes to the ~320 nm region. This leads to the apparent inversion of the band intensities at 310 nm (mainly tpy-based) and 320 nm [4,25,29,30].

In addition to this, the absorption bands around 400 nm are known to increase in intensity when the nitrogen atoms of the dppn-like fragment engage in hydrogen bonding with water [31]. Upon addition of DNA, the intensity of these bands decreases (see inset of Figure 4, left panel)—bands that are already more intense in water than in acetonitrile (see Figure 3 and Figure 4)—indicating that this portion of the ligand becomes shielded from interaction with water upon intercalation [1,3,31].

**Figure 5:**
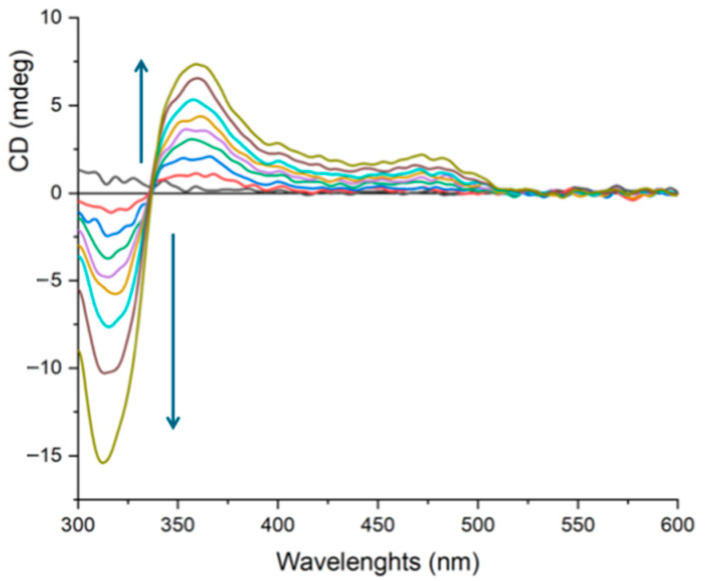
CD spectra of **1** (35 μM) with increasing amounts of CT-DNA, at 25 °C and pH 7 (1 mM phosphate buffer and 57 mM NaCl).

These combined spectroscopic changes are widely recognized as spectral fingerprints of a non-covalent intercalative binding mode [1,2,6,7,8]. More specifically, the extent of the observed hypochromism and the magnitude of the red-shift are consistent with the strong binding affinities typically observed for highly effective intercalating agents. The existence of a non-covalent type of interaction is also strongly suggested by the appearance, upon addition of DNA, of induced circular dichroism signals in the spectral region where only **1** absorbs (Figure 5).

These signals, due to the rigid orientation assumed by the complex bound to the chiral double helix, are also reversible by addition of sodium chloride, similarly to what already found in the spectrophotometric titration, thereby confirming the reversibility of the process. In the intercalation mechanism, the planar aromatic moiety of the ligand (in this case, the extended pydppn ligand) inserts itself directly between adjacent base pairs of the DNA double helix. This insertion often leads to a local unwinding and lengthening of the DNA helix to accommodate the intercalator [5,6]. The strong π−π stacking interactions between the intercalating ligand and the DNA base pairs, along with electrostatic interactions with the negatively charged DNA backbone, stabilize the complex-DNA adduct, which reflects in an increase in the thermal denaturation temperature of the biopolymer. At ionic strength 3 mM (2 mM in NaCl and 1 mM in phosphate buffer at pH 7), **1** causes an increase in DNA melting temperature (Δ*T*_m_ = 8.9 ± 0.5 °C) comparable to that of the well-known intercalator [Ru(bpy)_2_(dppz)]^2+^ [1] (Δ*T*_m_ = 13.2 ± 0.6 °C) and of [Os(tpy)(pydppz)]^2+^ (Δ*T*_m_ = 10.1 ± 0.5 °C), while [Ir(tpy)_2_]^3+^, which cannot intercalate [33], produces only a small increase (Δ*T*_m_ = 2.8 ± 0.4 °C). As further evidence of the intercalating ability of the Os(II) complex between the DNA base pairs, a viscometric titration of **1** with increasing amounts of rod-like DNA was performed. In fact, the elongation and simultaneous stiffening of the helix caused by a species intercalated between the nucleobases are reflected in an increase in the viscosity of the biopolymer solution [34].

**Figure 6:**
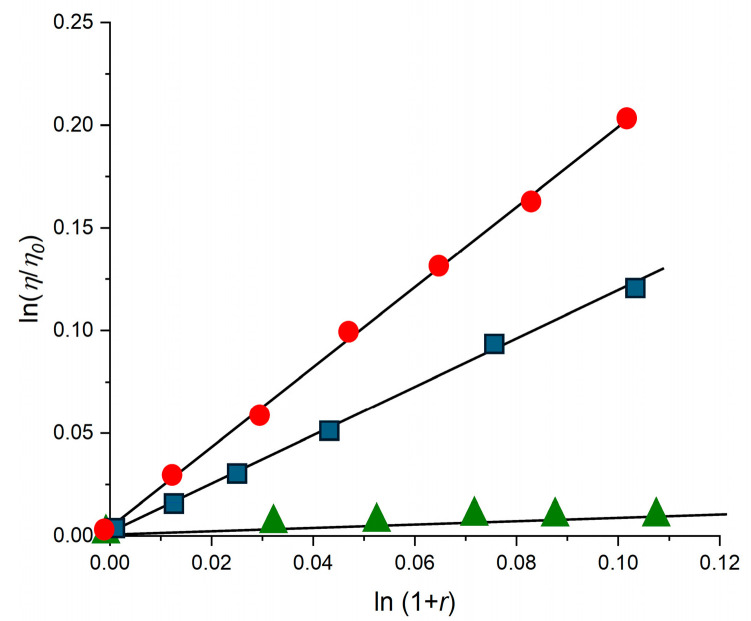
Viscometric titrations of CT-DNA solutions with **1** (blue squares), [Ru(bpy)_2_(dppz)]^2+^ (red circles), or [Ir(tpy)_2_]^3+^ (green triangles). The experiments were performed at 25 °C and pH 7 (1 mM phosphate buffer and 10 mM NaCl). In the axis labels, *η*_0_: reduced viscosity of the DNA solution in the absence of complex; *η*: reduced viscosity of the DNA solution in the presence of complex; *r* = [complex]_bound_/[DNA]_tot_.

As can be seen in Figure 6, the increases in viscosity of a rod-like DNA solution (about 600 ÷ 800 base pairs long) in the presence of increasing amounts of **1** are comparable to those induced by [Ru(bpy)_2_(dppz)]^2+^, while upon the addition of increasing amounts of [Ir(tpy)_2_]^3+^, the viscosity of the solution remains nearly constant. The collective spectrophotometric and emission data provide, together with the trend of the viscometric titration, compelling evidence that [Os(tpy)(pydppn)]^2+^ binds to CT-DNA primarily via an intercalative mechanism. Moreover, the fitting of spectrophotometric titration data of **1** with DNA gives an estimation of the binding constant value of the interaction through the McGhee von Hippel equation [35]. At ionic strength 58 mM (57 mM in NaCl and 1 mM in phosphate buffer at pH 7) *K*_B_ was found to be about 1.2 (±0.1) × 10^6^ M^−1^.

**Figure 7:**
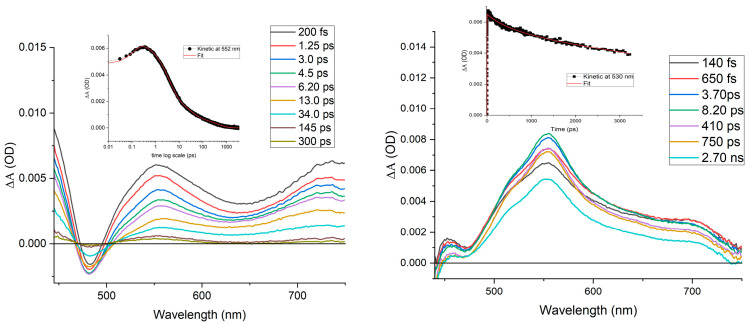
TASs of **1** (35 μM) at pH 7 (1 mM phosphate buffer and 57 mM NaCl) alone (**left**) and in the presence of an excess of CT-DNA (**right**); λ_exc_ = 400 nm. Inset: Fitting of selected traces.

As a further insight, pump–probe experiments were performed on both the bare **1** in phosphate buffer and on the complex intercalated into DNA. In Figure 7, the transient absorption spectra (TAS) of the Os(II) complex excited at 400 nm are reported. The initial transient absorption spectrum (black trace) of the complex exhibits a bleaching feature at 478 nm, indicative of metal-to-ligand charge transfer (MLCT) absorption, along with a prominent transient absorption band centered at 560 nm and a shoulder around 675 nm. The bleaching observed at 478 nm corresponds to the depletion of the ground-state absorption associated with the metal center, whereas the transient absorption at 560 nm is attributed to the reduced polypyridyl ligand. Comparison with literature data for analogous systems suggests that the species formed within the temporal window of excitation corresponds to the ^3^MLCT state, indicating that intersystem crossing occurs within less than 1 ps.

Within approximately 5 ps, the transient evolves into a new excited state, characterized by a more pronounced spectral contribution in the 625–750 nm region. This species undergoes vibrational relaxation over ~16 ps and decays to the ground state in under 400 ps. The transient formed at ~5 ps is likely associated with localization of the excited state on the phenazine moiety, stabilized by N–HOH hydrogen bonding, which accounts for the rapid non-radiative deactivation observed under steady-state conditions.

By contrast, **1** exhibits distinct behavior in the presence of DNA. The initially formed species (black trace), evolves over ~15 ps into a new transient, characterized by stronger absorption and increased spectral definition (band narrowing) in the 500–590 nm region, indicating vibrational cooling of the initially generated hot state. This relaxed state decays to the ground state on timescales exceeding the temporal resolution of our transient absorption setup, consistent with the excited-state lifetime measured by time-correlated single-photon counting (TCSPC).

These results point out the subtle interplay between ligand structure, solvent environment, and DNA interactions in determining the photophysical pathways and relaxation dynamics of polypyridyl metal complexes. A comprehensive understanding of these factors will be necessary for the eventual rational design of photoactive materials with predetermined properties for applications ranging from photochemistry to photophysics.

## 3. Materials and Methods

### 3.1. Chemicals

High-purity commercial reagent-grade products were used without further purification.

Calf thymus DNA was purchased from Sigma Chemical Co. (St. Louis, MO, USA) and purified as previously described [36]. The concentration, expressed in base pairs, was determined spectrophotometrically using the molar absorptivity [37]: 1.31 × 10^4^ M^−1^ cm^−1^ (260 nm). All the experiments with DNA were carried out at 25 °C and pH 7, in a phosphate buffer 1 mM and enough NaCl to give the desired ionic strength value.

### 3.2. Synthesis Procedures

**Synthesis of 3-(pirid-2′-il)-4,5,9,16-tetraaza-dibenzo[a,c]naftacene (pydppn)**

This compound was prepared according to the synthetic method reported by Turro et al. [4].

**Synthesis of [Os(tpy)(pydppn)](PF_6_)_2_ (1)**

[Os(tpy)Cl_3_] (46.9 mg, 0.093 mmol), prepared according to a literature procedure [38], was added to a suspension obtained by dissolving pydppn (49 mg, 0.121 mmol) in 3 mL of ethylene glycol. The reaction mixture was heated to reflux and stirred under a nitrogen atmosphere for 3 days. After cooling to room temperature, the resulting dark brown suspension was filtered, and the solid was washed with small portions of ethanol. The crude product was redissolved in a minimal amount of acetonitrile (CH_3_CN) and precipitated as the hexafluorophosphate salt upon addition of NH_4_PF_6_. The resulting brown-reddish solid was collected by filtration, dried under vacuum, and purified by column chromatography on silica gel using CH_3_CN/1 M NaNO_3_ (aq. sol.) (4:1) as the eluent. The dark brown complex [Os(tpy)(pydppn)](PF_6_)_2_ was obtained in 35% yield.

Elem. Anal. Calcd for C_42_H_26_F_12_N_8_OsP_2_: C, 44.93; H, 2.33; N, 9.98. Found: C, 44.11; H, 2.29; N, 9.81.

^1^H NMR (CD_3_CN): δ 10.05 (d, 1 H, *J* = 8.5 Hz), 9.75 (dd, 1 H, *J* = 1.1, 7.9 Hz), 9.40 (d, 1H, *J* = 8.7 Hz), 9.25 (dd, 2 H, *J* = 1.5, 4.4 Hz), 8.85 (d, 2 H, *J* = 8.5 Hz), 8.68 (d, 1 H, *J* = 7.8 Hz), 8.47 (m, 5 H), 8.10 (m, 1 H, *J* = 1.1, 7.8 Hz), 7.80 (m, 3 H, *J* = 1.2, 8.1 Hz), 7.60 (m, 4 H), 7.45 (m, 2 H, *J* = 5.1, 7.7 Hz), 7.23 (dd, 1 H, *J* = 0.9, 5.3 Hz), 7.06 (ddd, 2 H, *J* = 2.0, 5.0, 7.4 Hz).

### 3.3. Instrumentations

^1^H Nuclear Magnetic Resonance (NMR) spectroscopy was performed on a Bruker AMX R-300 spectrometer using deuterated acetonitrile (CD_3_CN) as solvent.

Electrochemical measurements were carried out in argon-purged acetonitrile at room temperature with an Metrohm Autolab PGSTAT12 (Utrecht, The Netherlands) equipment interfaced to a PC. The working electrode was a glassy carbon (8 mm^2^, Amel) electrode. The counter electrode was a platinum wire, and the quasi-reference electrode was a Ag wire (ferrocene was used as internal standard). The concentration of the solution was about 700 μM. Tetrabutylammonium hexafluorophosphate was used as supporting electrolyte and its concentration was 50 mM. Cyclic voltammograms were performed at scan rates of 20, 50, 200, and 500 mV s^−1^. For reversible processes, half-wave potentials (vs. SCE) were calculated as the average of the cathodic and anodic peaks. The criteria for reversibility were the separation of 59 mV between cathodic and anodic peaks, the close to unity ratio of the intensities of the cathodic and anodic currents, and the constancy of the peak potential on changing scan rate. The number of exchanged electrons was measured with differential pulse voltammetry (DPV) experiments performed with a scan rate of 20 mV s^−1^, a pulse height of 75 mV, and a duration of 40 ms, and by taking advantage of the presence of ferrocene used as the internal reference.

Absorption spectra were recorded with a Cintra 3030 GBC spectrophotometer (GBC Scientific, Keysborough, Australia).

Spectrophotometric titration was performed by adding to a complex solution (35 μM) successive aliquots of DNA, containing also the complex, in a 10 mm stoppered quartz cell and recording the spectrum after each addition. The data were analyzed by a nonlinear least-squares fitting program, applied to McGhee and von Hippel equation [31]. The binding constant, *K*_B_, was determined by the program, using the extinction coefficient of the compounds, the free complex concentration and the ratio of bound complex per mole of DNA. Extinction coefficient for bound complex was determined by Beer’s law plots in the presence of a large excess of DNA.

Luminescence spectra were recoded with a Spex-Jobin Yvon FluoroMax-2 (Palaiseau, France), equipped with a Hamamatsu R3896 photomultiplier (Hamamatsu City, Japan). The spectra were corrected for photomultiplier response using a program purchased with the fluorimeter. Luminescence lifetimes were determined by time-correlated single-photon-counting (TCSPC) with an Edinburgh OB900 spectrometer (light pulse: Hamamatsu PL2 laser diode, pulse width 59 ps at 408 nm) (Livingston Village, Scotland). Experimental uncertainties are as follows: absorption maxima, 2 nm; emission maxima, 5 nm; excited-state lifetimes, 10%, unless otherwise stated. For the quantum yield determination [Os(tpy)(pydppz)](PF_6_)_2_ was used as reference in acetonitrile (*Φ* = 0.008) [25].

Circular dichroism experiments were performed by a Jasco J-810 spectropolarimeter (Milano, Italy).

The thermal denaturation temperature of complex–DNA mixtures (1:10) was determined spectrophotometrically in 1 mM phosphate buffer solutions at pH 7 containing the compound (7.8 μM) and 2 mM NaCl. Melting curves were recorded at 260 nm. The temperature has been increased at a rate of 0.5 °C/min by using a GBC PTP-1 Peltier system (Keysborough, Australia).

Viscosity titrations were performed by means of a Cannon-Ubbelhode semi-micro-dilution viscometer—Series No. 75, Cannon Instrument Co.- (State College, PA, USA), thermostatically maintained at 25 °C in a water bath. The viscometer contained 2 mL of sonicated DNA solution, in 1 mM phosphate buffer (pH = 7) and 10 mM NaCl. The compound solution (100 ÷ 250) μM, containing also DNA (600 μM) at the same concentration as that in the viscometer, was delivered in increments of 90 ÷ 570 µL from a micropipette. Solutions were freed of particulate material by passing them through Acrodisc syringe filters before use. Flow times were measured by hand with a digital stopwatch. Reduced viscosities were calculated by established methods and plotted as ln *η*/*η*^0^ against ln (1 + *r*) for rodlike DNA (600 base pairs) (*η* = reduced viscosity of the DNA solution in the presence of complex; *η*^0^ = reduced viscosity of the DNA solution in the absence of complex; *r* = [complex]_bound_/[DNA]_tot_).

Time-resolved transient absorption experiments were performed using a pump–probe setup based on the Spectra-Physics MAI-TAI Ti:sapphire system (Campagnano di Roma, Italy) as the laser source and the Ultrafast Systems Helios spectrometer (Sarasota, FL, USA) as the detector. The pump pulse was generated using a Spectra-Physics 800 FP OPA instrument (Campagnano di Roma, Italy). The probe pulse was obtained by continuum generation on a sapphire plate (spectral range 450–800 nm). The effective time resolution was around 200 fs, and the temporal chirp over the white-light 450–750 nm range around 150 fs; the temporal window of the optical delay stage was 0–3200 ps. In order to cancel out orientation effects on the dynamics, the polarization direction of the linearly polarized probe pulse was set at a magic angle of 54.7° with respect to that of the pump pulse. Please note that all the transient spectra shown in the present paper are chirp corrected. This correction was done by using the pump induced absorption signals themselves in the same conditions (same cuvette, solvent, temperature, stirring frequency…) used for each single experiment. All the timeresolved data were analyzed with the Ultrafast Systems Surface Xplorer Pro version 4.5 software.

## 4. Conclusions

The work’s findings demonstrate the remarkable potential of [Os(tpy)(pydppn)]^2+^ as a molecular probe that intercalates into DNA. Its unique luminescence response allows for sensitive detection of nucleic acids. Osmium-based architectures are advantageous for applications that benefit from selective DNA interaction and extended optical penetration because of their gainful photophysical characteristics, which include absorption and emission in the near-infrared region.

[Os(tpy)(pydppn)]^2+^ is a promising prototype for the logical design of next-generation metal-based systems with adjustable photophysical behavior and possible light-activated therapeutic relevance, according to these findings. All things considered, this research offers a strong basis for future investigation into osmium complexes as multipurpose substances with both therapeutic and diagnostic properties.

## Figures and Tables

**Table 1:** Redox data for **1** and **MOD** in CH_3_CN ^[a]^.

	E_1/2_ ^ox^/V vs. SCE	E_1/2_ ^red^/V vs. SCE
**1**	+0.99 [1]	−0.67 [1]; −1.08 [1]; −1.32 [1]; −1.56 [1]; −1.83 [1]
**MOD** ^[b]^	+0.89 [1]	–0.88 [1]; –1.21 [1]; –1.36 [1]; –1.53 [1]

^[a]^ The number of electrons exchanged is given in square brackets. Experimental error: ±10 mV. ^[b]^ Data from ref. [25].

**Table 2:** Spectroscopic and photophysical data for **1** and **MOD** in CH_3_CN.

	Absorption	Luminescence		
	λ_max_ [nm](ε [M^−1^cm^−1^])	λ_max_ [nm]	*Φ*	τ [ns]
**1**	328 (54,100) 473 (11,900)	830	0.02	110
**MOD**	325 (50,500) 375 (22,100) 475 (14,600)	800	0.008	50

## Data Availability

Data are contained within the article and Supplementary Materials.

## Supplementary Materials

The following supporting information can be downloaded at: https://www.mdpi.com/article/10.3390/molecules30244671/s1, Figure S1: Absorption spectra of 1 in 1 mM phosphate buffer (pH = 7) and 10 mM NaCl, before and after thermal treatment.; Figure S2: Absorption spectra of 1 in acetonitrile before and after thermal treatment; Figure S3. Absorption and emission spectra of the reference and 1 in acetonitrile for the determination of the quantum yield. Ref. [39] is cited in the Supplementary Materials.
