# Supplementary material for: Lighting Up DNA in the Near-Infrared: An Os(II)–pydppn Complex with Light-Switch Behavior
Source: Molecules. 2025 Dec 5;30(24):4671. doi: 10.3390/molecules30244671 (PMC12736211; doi:10.3390/molecules30244671)
Supplement: Supplementary file 1 [file molecules-30-04671-s001.zip › molecules-4009969-supplementary.pdf]

molecules

MDPI

Supplementary Materials

Lighting Up DNA in the Near-Infrared: An Os(II)–pydppn Complex with Light-Switch Behavior

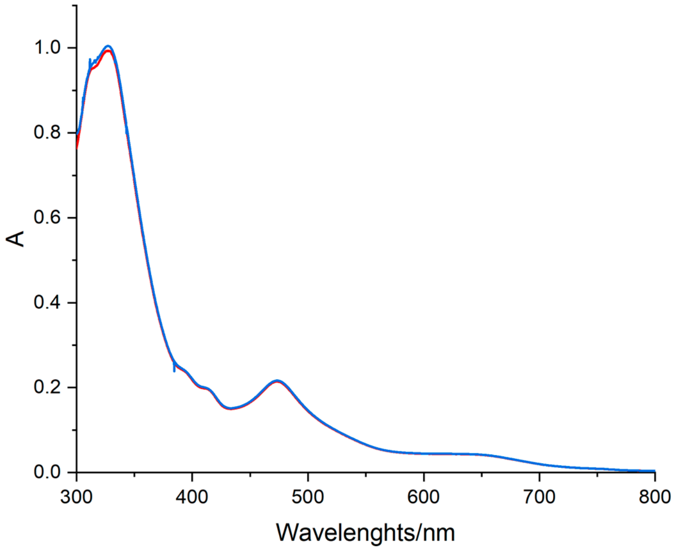

Figure S1. Absorption spectra of **1** in 1 mM phosphate buffer (pH = 7) and 10 mM NaCl, before and after thermal treatment. The red trace corresponds to the spectrum recorded after keeping the sample at 45 °C for two days under continuous stirring in a sealed cuvette to prevent solvent evaporation. The post-treatment spectrum was collected after cooling the sample back to room temperature.

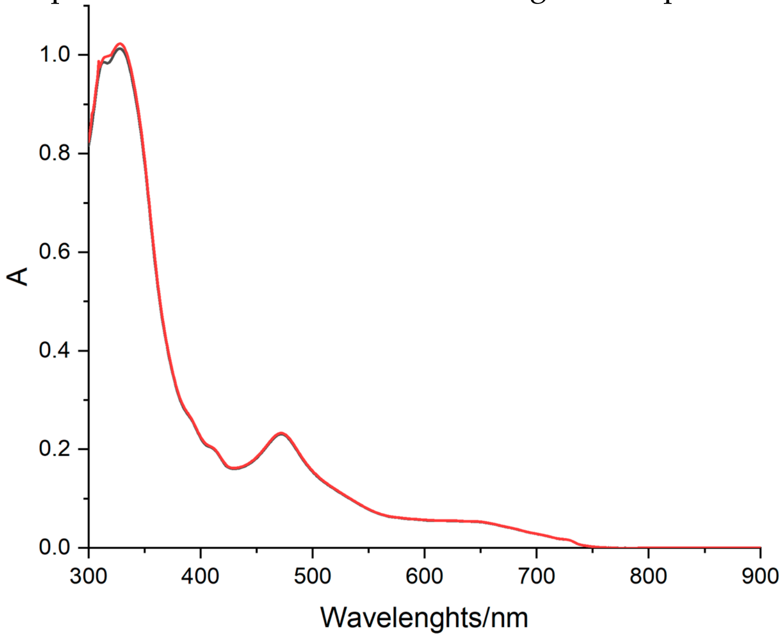

Figure S2. Absorption spectra of **1** in acetonitrile before and after thermal treatment. The red trace corresponds to the spectrum recorded after keeping the sample at 45 °C for two days under continuous stirring in a sealed cuvette to prevent solvent evaporation. The post-treatment spectrum was collected after cooling the sample back to room temperature.

Quantum yield

Emission quantum yields for deaerated solutions were determined using the optically diluted method [39]. As luminescence quantum yield standards, we used $[Os(tpy)(pydppz)](PF_6)2$ as reference in acetonitrile ($\Phi = 0.008$) [25].

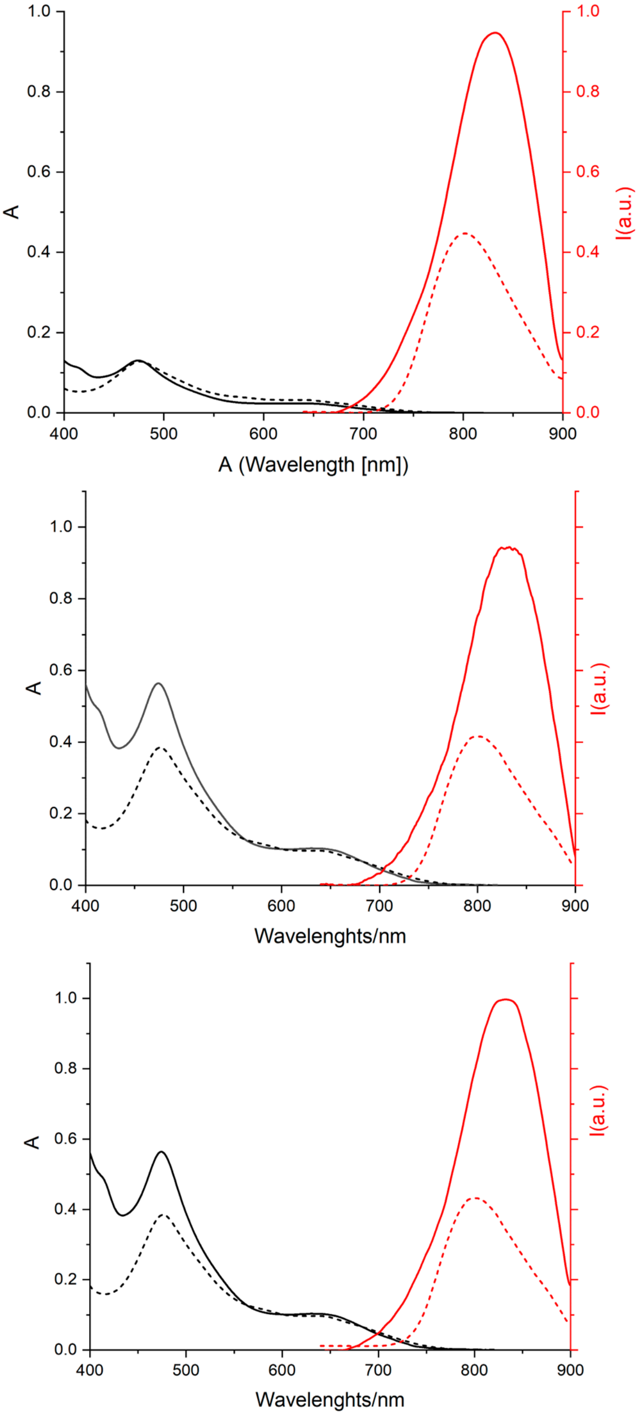

Figure S3. Absorption (black lines) and emission (red lines) spectra of the reference (dashed lines) and **1** (full lines) in acetonitrile for the determination of the quantum yield. Both samples were excited at 475 nm (top panel), 600 nm (middle panel), and 620 nm (bottom panel).
